# Ellagic Acid Controls Cell Proliferation and Induces Apoptosis in Breast Cancer Cells via Inhibition of Cyclin-Dependent Kinase 6

**DOI:** 10.3390/ijms21103526

**Published:** 2020-05-15

**Authors:** Mohd Yousuf, Anas Shamsi, Parvez Khan, Mohd Shahbaaz, Mohamed F. AlAjmi, Afzal Hussain, Gulam Mustafa Hassan, Asimul Islam, Qazi Mohd Rizwanul Haque, Md. Imtaiyaz Hassan

**Affiliations:** 1 Microbiology Research Laboratory, Department of Biosciences, Jamia Millia Islamia, Jamia Nagar, New Delhi 110025, India; yousufbiochem@gmail.com (M.Y.); qhaque@jmi.ac.in (Q.M.R.H.); 2 Centre for Interdisciplinary Research in Basic Sciences, Jamia Millia Islamia, Jamia Nagar, New Delhi 110025, India; anas.shamsi18@gmail.com (A.S.); parvezynr@gmail.com (P.K.); aislam@jmi.ac.in (A.I.); 3 South African Medical Research Council Bioinformatics Unit, South African National Bioinformatics Institute, University of the Western Cape, Private Bag X17, Bellville, Cape Town 7535, South Africa; mohammed.shahbaaz@gmail.com; 4 Laboratory of Computational Modeling of Drugs, South Ural State University, 76 Lenin Prospekt, 454080 Chelyabinsk, Russia; 5 Department of Pharmacognosy, College of Pharmacy, King Saud University, Riyadh 11451, Saudi Arabia; malajmii@ksu.edu.sa (M.F.A.); afzal.hussain.amu@gmail.com (A.H.); 6 Department of Biochemistry, College of Medicine, Prince Sattam Bin Abdulaziz University, P.O. Box 173, Al-Kharj 11942, Saudi Arabia; mgulam@gmail.com

**Keywords:** cyclin-dependent kinase 6, natural products, ellagic acid, molecular dynamics simulation, kinase inhibitor, drug design and discovery, anticancer therapy

## Abstract

Cyclin-Dependent Kinase 6 (CDK6) plays an important role in cancer progression, and thus, it is considered as an attractive drug target in anticancer therapeutics. This study presents an evaluation of dietary phytochemicals, capsaicin, tocopherol, rosmarinic acid, ursolic acid, ellagic acid (EA), limonene, caffeic acid, and ferulic acid for their potential to inhibit the activity of CDK6. Molecular docking and fluorescence binding studies revealed appreciable binding affinities of these compounds to the CDK6. Among them, EA shows the highest binding affinity for CDK6, and thus a molecular dynamics simulation study of 200 ns was performed to get deeper insights into the binding mechanism and stability of the CDK6-EA complex. Fluorescence binding studies revealed that EA binds to the CDK6 with a binding constant of *K* = 10^7^ M^−1^ and subsequently inhibits its enzyme activity with an IC_50_ value of 3.053 µM. Analysis of thermodynamic parameters of CDK6-EA complex formation suggested a hydrophobic interaction driven process. The treatment of EA decreases the colonization of cancer cells and induces apoptosis. Moreover, the expression of CDK6 has been downregulated in EA-treated human breast cancer cell lines. In conclusion, this study establishes EA as a potent CDK6 inhibitor that can be further evaluated in CDK6 directed anticancer therapies.

## 1. Introduction

Pathological stages of cancer cells are characterized by dysregulated cell proliferation, increased cell survival ability, loss of differentiation, accumulation of genetic mutation, and alteration in the metabolic pathways [1]. Uncontrolled cell division and alteration in metabolism are major hallmarks of cancer [2]. Cancer therapeutics generally involves the identification of potential targets that play a direct or indirect role in the cell division and regulation of cell metabolism. Protein kinases regulate >80% of cellular pathways namely cell cycle progression, transcription, DNA repair, and metabolic events in different signaling cascades through phosphorylation/dephosphorylation and thus used as a potential drug target in cancer therapy [3].

Cyclin-dependent kinases (CDKs) belongs to serine/threonine-protein kinases and plays an important role in cell cycle progression [4]. The catalytic activities of CDKs are modulated by their interactions with cyclins and CDK inhibitors (CKIs) [5]. Every stage of the cell cycle is strictly controlled by CDKs and respective cyclins and CKIs and close cooperation between these ensure orderly progression of the cell cycle [6]. Based on the sequence similarity of the kinase domain, CDKs are categorized as a member of the CMGC group of kinases [7]. *CDK6* gene is located on chromosome 7q21.2, encodes a 326 amino acids monomeric protein [8], which is involved in the regulation of key metabolism and cell cycle [9]. CDK6 and cyclin D is responsible for G1 to S phase transition during the cell division. Retinoblastoma (RB), a tumor suppressor, plays a switching role in the cell cycle [10]. E2F is an evolutionarily conserved family of factors that control cell cycle and significantly contributes in tumor development [6]. CDK6 is involved in the (RB)-E2F signaling and subsequently cancer progression. Uncontrolled regulation of the cyclin D-CDK4/6-INK4-RB pathway is observed in cancer resulted from an uncontrolled cell cycle and cell growth [11]. CDK6 regulates glucose metabolism by inhibiting the activity of phosphofructokinase (PFK) and pyruvate kinase (PKM) of glycolysis through phosphorylation and subsequently reduces the production of reactive oxygen species (ROS) in cells [9]. ROS are very toxic to a cell and cause apoptosis. Nonselective CDK inhibitors failed in the clinical trials due to their toxic effect on non-cancer cells. Thus, the identification of a selective CDK-6 inhibitor may be used as therapeutic targeting of cancer cell growth and metabolic alterations [2,9].

Flavonoids are generally present in different parts of plants and are of great clinical significance [12,13]. These phytoconstituents target several pathways, affect glucose homeostasis, and control cell growth. For the past few decades, phytoconstituents of medicinal plants have been extensively used for the treatment of cancer with minimal side effects. Plant-derived formulations and phytochemicals are extensively used to manage different diseases [14]. In recent years, many phytoconstituents are recommended as potent kinase inhibitors to control cell growth and metastasis [15,16,17,18]. Plant-derived products possess anti-oxidant, anti-cancerous, anti-inflammatory, anti-diabetic, anti-microbial, and hepato-protective features [19].

This study reports the binding efficiency and mechanism of interaction of ellagic acid (EA) to the CDK6 using a combined in silico and in vitro assays. Molecular docking was performed to dissect the mode of interaction of EA with CDK6. Molecular dynamics (MD) simulations investigated the stability of the CDK6-EA system. Enzyme assay suggested significant inhibition of CDK6 by EA. Further, fluorescence binding studies, complemented by the isothermal titration calorimetry (ITC), suggested that EA binds to CDK6 with excellent affinity and a stable CDK6-EA complex is formed. Cell-based expression and therapeutic evaluation showed that EA treatment decreases the expression of CDK6, inhibited cell proliferation, and induces apoptosis in the breast cancer cells.

## 2. Results and Discussion

### 2.1. Cloning, Expression, and Purification of CDK6

The *CDK6* gene from plasmid pcDNA was amplified by PCR, with *Nco*I and *Xho*I site at the 5′ and 3′ ends, respectively. The size of the amplified product was 981bp (Figure S1A). It was digested with *Nco*I and *Xho*I and subsequently ligated into pET28a^+^ backbone digested with similar restriction enzymes. The ligated product was transformed into *E. coli* and positive colonies were selected and confirmed by colony PCR (Figure S1B). The positive clones were confirmed with the help of restriction digestion using *Nco*I and *Xho*I endonucleases (Figure S1C). Finally, the constructed plasmid was verified by DNA sequencing. The confirmed plasmid construct pET28a^+^ with CDK6 was transformed into *E. coli BL21* (codon^+^). The recombinant protein was expressed at 18 °C by inducing it with 0.5 mM IPTG for 20 h. The over-expression of CDK6 was observed using SDS-PAGE with an apparent molecular weight of ~37 kDa (Figure S2A). The CDK6 was purified using Ni-NTA chromatography (Figure S2B). A single band was observed on the SDS-PAGE confirms the purity of CDK6. The purified CDK6 was finally confirmed using Western blot (Figure S2C). The purified protein was subjected to absorption spectrum measurements in the range of 240–340 nm, which revealed proper folding of purified CDK6 without any aggregation (Figure S3).

### 2.2. Docking-Based Screening of Natural Compounds

**Figure 1:**
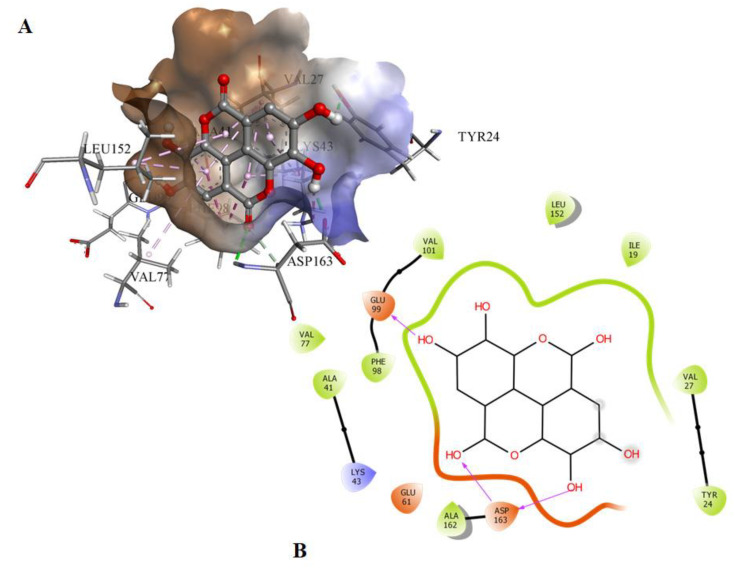
(**A**) The docked conformation of EA and CDK6 in the 3-D view showing the bound orientation of the inhibitor. (**B**) The 2-D view of the docked complexes highlighting the observed hydrogen bonds between the inhibitor and protein molecules.

Molecular docking was used to screen a series of natural compounds, limonene, ursolic aid, caffeic acid, ferulic acid, tocopherol, capsaicin, and EA to select the best inhibitors of CDK6. Binding affinities and interactions of ligand to functionally important residues of CDK6 were calculated by docking and subsequently in vitro fluorescence binding studies were performed to select the best possible inhibitor of CDK6 (Table SI). Information about the active site of CDK6 was obtained from the published report [20]. The ATP-binding site of CDK6 is predominated by the Lys43, Phe98, His100, Asp104, Thr107, and Asp163. EA binds to the same pocket with three hydrogen bonds offered by Glu99 and Asp163 (Figure 1). The oxygen of the carbonyl group of Glu99 forms the hydrogen bond with the H28 of the EA with the electropositive characteristic provided by the O6 atom present in the vicinity. In contrast to Glu99, the hydrogen bonding of Asp163 was observed with the side chain in which interaction was observed with the O1 and O3 of the EA. The 22nd pose was selected based on the scoring of the docked poses with a free energy of binding of −6.25 kcal/mol and inhibition constant of 26.35 µM. These results suggested that EA binds strongly to the active site pocket of CDK6 and subsequently inhibits its activity. The selected docked system was subjected to MD simulations for further analysis.

### 2.3. MD Simulations

**Figure 2:**
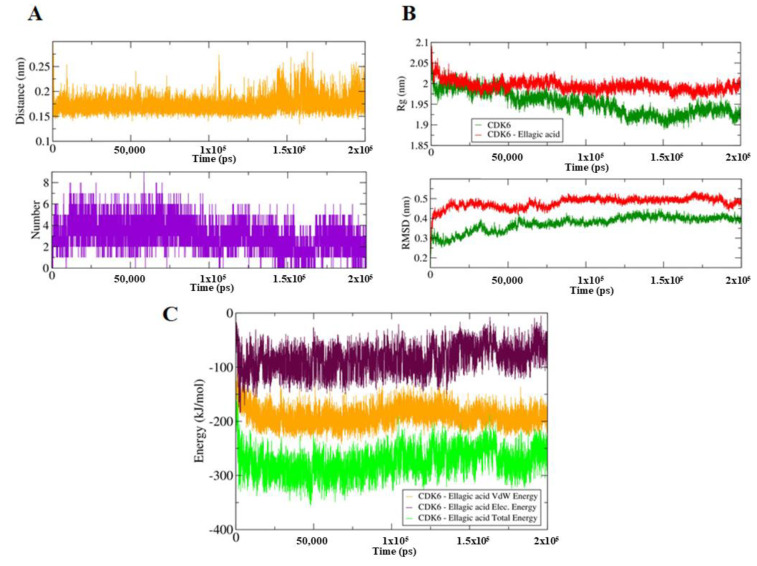
(**A**) Upper panel shows a plot of changes in the distance between the CDK6 and EA. The lower panel shows the number of hydrogen bond fluctuation curve highlighting the changes in the observed number. (**B**) The upper panel shows the *R*_g_ curves, an indication of compactness. The lower panel corresponds to the RMSD plots to highlight the conformational changes in the CDK6 upon EA binding. (**C**) The MMPBSA-based generated curves showing changes in the total, electrostatic, and van der Waals energies between the CDK6 and CDK6-EA complex.

To identify the impact of EA binding on the conformation of CDK6, we have performed MD simulation for both EA-bound and unbound forms of CDK6 on a time scale of 200 ns (Total 400 ns). EA binding induces a closed conformation of CDK6, which is indicative of the computed distance of 0.15–0.3 nm as well as from the number of existed hydrogen bonds up to nine between the CDK6 and EA during MD simulations (Figure 2A). The stability of the system was further assessed using the calculated radius of gyration (*R*_g_) and root mean square deviation (RMSD) values, which showed that the system achieved an equilibrium after 120 ns (Figure 2B). There is a visible difference in the *R*_g_ and RMSD values between the EA and bound and unbound forms of CDK6. We noticed that the bound form is slightly less stable than the unbound form of CDK6, indicating a perturbation in the structure after EA binding.

**Figure 3:**
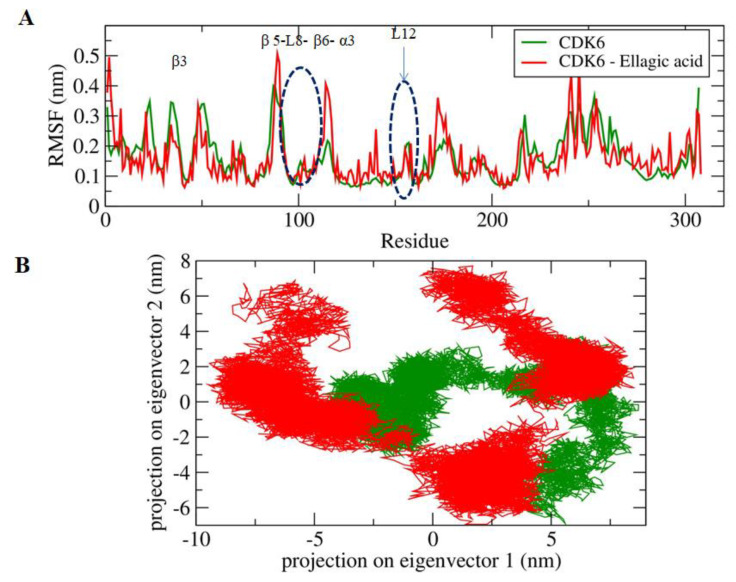
(**A**) The graphical representation of the changes observed in the fluctuation of the constituent residues between the EA bound and unbound CDK6. (**B**) The 2-D eigenvector projection plot showing the differences between the flexibility of the two studied forms.

The active site of the CDK6 is occupied by Lys43, Phe98, His100, Asp104, Thr107, and Asp163. A significant difference in the fluctuations of constituent residues of EA-bound and unbound conformations of CDK6 was observed (Figure 3A). The active site residues form β3, β5, L8, β6, α3, and L12 regions of the secondary structure elements showed relatively lower motion in the constituent residues in the unbound form of the CDK6, which indicated the presence of lower relative energy as compared to the bound form. Furthermore, the flexibility of the conformational states of both EA-bound and unbound forms were assessed using Principle Component Analysis (PCA), a statistical technique used for the reduction of the data complexity and is significant in assessing the variation in the atomic motion in biomolecules in the course of MD simulation. A set of eigenvectors and eigenvalues were used to describe the motion of the protein atomic structure. The PCA is significant in the established correlation between the protein functionalities and conformation. The EA-bound form occupies a larger conformational space as compared to the unbound conformation of the CDK6 (Figure 3B), indicating the occurrence of higher structural stability in the unbound form as compared to the bound form. 

**Figure 4:**
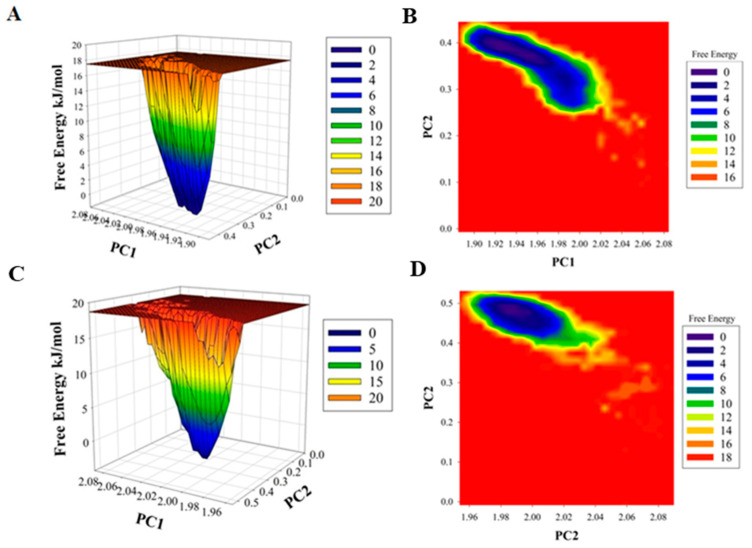
Plots of (**A**) free energy landscape and (**B**) Contour map for the of CDK6. A graphical representation of the (**C**) free energy landscape and (**D**) Contour map for the of CDK6-EA.

The difference in the folding pattern between the bound and the unbound form of CDK6 was further studied by analyzing the free energy landscapes of the two conformations. A distinct difference in the free energies was observed between the two conformations (Figure 4). In the free CDK6, favored energy and relatively stable conformation were observed (Figure 4A,B) as compared to the EA-bound form, indicating that EA-binding to CDK6 perturbs its folding pattern and inhibits the functionality of the protein. Further, the MMPBSA-based algorithms were used for the calculation of the energies present between the CDK6 and EA in the docked complexes (Figure 4C,D). The total free energy of binding between the protein and the inhibitor was observed between −300 to −350 kJ/mol with electrostatic energy as the major contributor to the binding of the EA with CDK6. This observation validated the reliability of the EA-binding to the CDK6.

### 2.4. Binding Affinity and Thermodynamics of CDK6-EA Interaction

**Figure 5:**
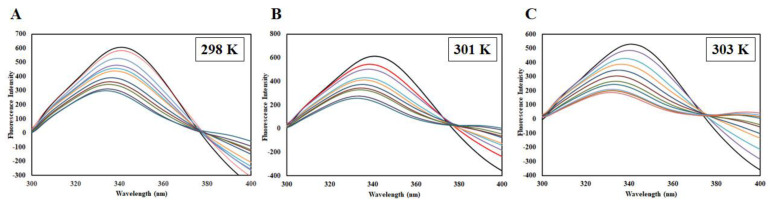
Steady-state fluorescence of CDK6 in the absence and presence of EA (1–10 µM) at (**A**) 298 K, (**B**) 301 K, and (**C**) 303 K.

To further complement our in-silico studies, fluorescence spectra measurements were carried out. Intrinsic fluorescence of a protein is an indicator of changes in the microenvironment of buried tryptophan upon ligand binding. Upon ligand binding, fluorescence quenching occurs, which is defined as a reduction in the fluorescence intensity with a subsequent rise in the ligand concentration [21]. This quenching can either be static or dynamic or a combination of both. As we increase the EA concentration, a significant decrease in the fluorescence intensity of CDK6 was observed, indicating an excellent binding affinity, *K* = 2.6 × 10^7^ M^−1^ (Figure 5).

**Figure 6:**
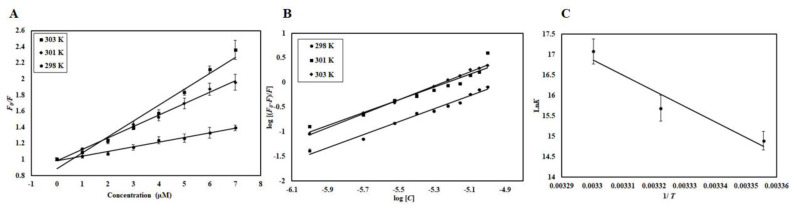
(**A**) Stern-Volmer plots, (**B**) Modified Stern-Volmer plots, and (**C**) van ’t Hoff plots of CDK6-EA interaction at three temperatures (298 K, 301 K, and 303 K).

EA binding to the CDK6 causes an alteration in the Trp microenvironment that causes a decline in the fluorescence intensity, which was mathematically fitted to estimate binding parameters. A change in such parameters as a function of temperature can be used to differentiate between static and dynamic modes of quenching [22]. Fluorescence intensity was measured at three different temperatures (298, 301, and 303 K). A decrease in the fluorescence intensity with increasing EA concentration was fitted into the modified Stern-Volmer equation, a plot of *F*_0_/*F* v/s [*C*]; the slope of the plot giving Stern-Volmer constant *K*_sv_ at fixed intercept after linear regression (Figure 6A). For the calculation of *K*_sv_, only linear points were taken into consideration. If there is a linear dependence between *F*_0_/*F* v/s [*C*], it is suggesting the existence of only one mode of quenching, either static or dynamic because the characteristic Stern-Volmer plot of combined quenching (both static and dynamic) is an upward curvature. Table 1 listed the values of obtained *K*_sv_ at three different temperatures. We found that *K*_sv_ increases with a corresponding increase in temperature. The *K*_sv_ value decreases with increasing temperature for static quenching as a result of complex formation, which undergoes dissociation by increasing the temperature while the reverse effect is observed for dynamic quenching, where *K*_sv_ increases with temperature because of higher temperature resulting in faster diffusion of the quencher and hence larger extent of collisional quenching [22]. Thus, we concluded that dynamic quenching governs the CDK6-EA complex formation.

The mode of quenching was further validated from the value of the bimolecular quenching rate constant, *K_q_*, listed in Table 1. The value of *K_q_* for CDK6-EA interaction was higher than the maximum scatter collision quenching constant of different quenchers with biopolymers (2 × 10^10^ M^−1^s^−1^) suggesting a static mode of quenching to be operative in the case of CDK6-EA binding. Based on these observations, we concluded that CDK6-EA quenching is guided by a mixture of both static and dynamic modes of quenching.

The modified Stern-Volmer equation was employed to estimate binding constant (*K*) and the number of binding sites (*n*). Figure 6B shows experimental data fitting as per the modified Stern-Volmer equation; the intercept of this plot gives the value of *K* while the slope gives the number of binding sites, *n*. Table 2 shows the *K* obtained at different temperatures and it was found that a more stable complex is formed at higher temperatures evident from the increase in *K* with a corresponding temperature rise. The value of binding constant (*K*) was found to be 2.6 × 10^7^ M^−1^ at 303 K, indicating a very strong interaction between CDK6 and EA as this range is reported for other complexes [23,24]. Fluorescence-based studies further validated our previous observations and suggested a strong interaction between CDK6 and EA. 

Electrostatic, hydrogen bonds, van der Waals, and hydrophobic interactions are major forces involved in protein-ligand interactions. The magnitudes and signs of thermodynamic parameters (Δ*H*°, Δ*S*°, and Δ*G*°) are used to understand which forces govern the reaction. Equation (1) was used to find all thermodynamic parameters associated with CDK6-EA interaction. Figure 6C shows the van ’t Hoff plot shows the dependence of binding constant (*K*) on 1/*T*, the slope of which is equal to −ΔH/R, and the intercept gives an estimate of Δ*S*/R. Table 2 depicts all the thermodynamic parameters obtained for CDK6-EA interaction. When the values of Δ*H*° and Δ*S*° are negative, the dominant forces are van der Waals force and hydrogen bonding, while the positive values of Δ*H*° and Δ*S*° mark the existence of hydrophobic interactions [25]. For CDK6-EA interaction, positive values of Δ*H*° and Δ*S*° suggested that interaction was driven by dominant hydrophobic interactions.

### 2.5. Isothermal Titration Calorimetry

**Figure 7:**
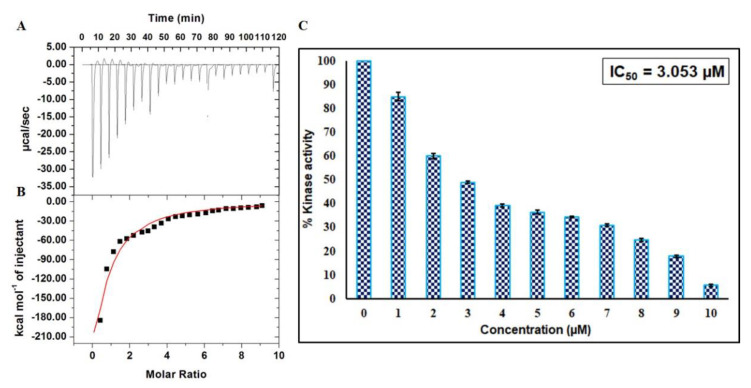
Binding and enzyme inhibition studies of EA with CDK6: (**A**) Representative thermogram showing the heat of dilutions of EA with CDK6. (**B**) Binding isotherm for the titration of EA with CDK6 at 25 °C. The molar concentration of CDK6 in the sample cell was 15 μM. EA molar concentration in the syringe was 500 μM. (**C**) ATPase inhibition assay of CDK6 with increasing concentration of EA (0–10 µM). The activity of native CDK6 was taken as 100% for reference.

An ITC experiment was performed to estimate thermodynamic parameters like change in entropy (Δ*S*), number of binding sites (*n*), Gibbs free energy (Δ*G*), change in enthalpy (Δ*H*), and binding constant (*K_a_*) to further complement fluorescence binding studies [26]. A representative calorimetric isotherm is shown in Figure 7A, in which every peak corresponds to a single round of injection of EA into the CDK6. Figure 7B shows the enthalpy change with each injection as a function of the molar ratio of EA into CDK6. This data was obtained for the model of one binding site. The thermodynamic parameters obtained for CDK6-EA interaction were: *K*_a_ = 2.52 × 10^4^ ± 8.8 × 10^3^ M^−1^, ∆*H* = −1.18 × 10^8^ ± 2.25 × 10^10^ kcal mol^−1^, and ∆*S* = −3.99 × 10^5^ cal.mol^−1^K^−1^. Interestingly, there is a noticeable difference in the thermodynamic parameters estimated using ITC and fluorescence, but such variations were observed earlier in other studies due to the assumptions made in noncalorimetric approaches that Δ*H* does not depend on temperature [27,28]. Furthermore, ITC interprets an overall change in a particular parameter while fluorescence spectroscopy measures a local change in the Trp.

### 2.6. Enzyme Inhibition Assay

To investigate the consequence of EA on the kinase activity of CDK6, enzyme activity assay was carried out with increasing concentrations of EA (1–10 µM) (Figure 7C). Enzyme activity was performed as per our previously published protocols [29,30]. It is quite apparent from the inhibition data that the activity of CDK6 decreases with increasing concentration of EA and shows a concentration-dependent relationship. IC_50_ was calculated with an AAT Bioquest calculator [31] (Figure S3C) and was found to be 3.053 µM. These observations suggested that EA is a strong inhibitor of CDK6.

### 2.7. Cell Viability Studies

**Figure 8:**
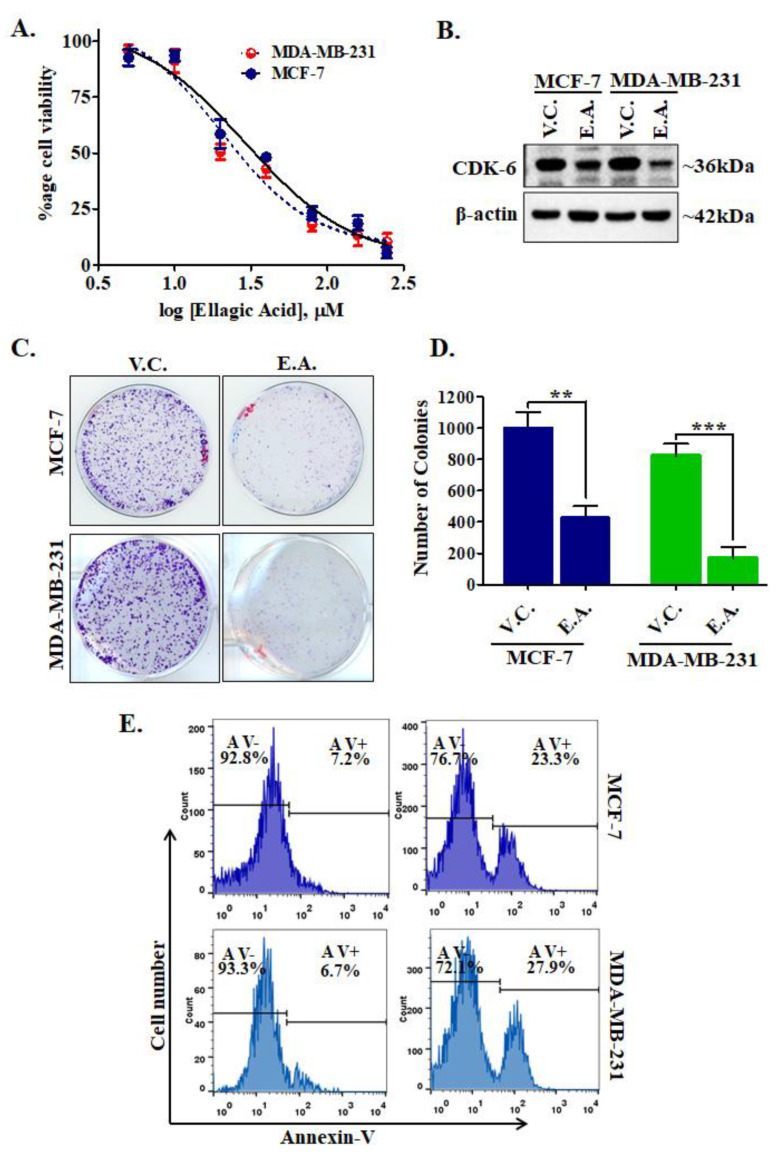
Treatment of EA decreases cell viability, CDK6 expression, colonization, and induces apoptosis in cancer cells. (**A**) The cell proliferation profile showing the effect of EA treatment on the viabilities of MCF-7 and MDA-MB-231 cells. The selected cancer cells were incubated with an increasing dose of EA for 48 h and respective cell viabilities were estimated using MTT assay. Each data point shown is the mean ± SD from *n* = 3. (**B**) Protein expression of CDK6 in EA and vehicle-treated MCF-7 and MDA-MB-231 cells. (**C**) Colony formation assay performed with respective IC_50_ concentrations of EA with selected cell lines. (**D**) The bar graph representation for the total number of colonies in each treatment group and compared with the vehicle controls. (**E**) Apoptosis studies showing the percentage of Annexin-V positive/negative cells stained after EA treatment for 48 h. The data were statistically analyzed using Student’s *t*-test for unpaired samples, ** *p* < 0.01, *** *p* < 0.001 compared to the vehicle control group.

CDK6 is an important drug target for varying types of cancers, but most prominently used for the targeting of breast cancer [32,33]. We have evaluated the effect of EA treatment on the cell viabilities of MCF-7 and MDA-MB-231 human breast cancer cell lines. Cell viability studies showed a significant decrease in the proliferation of selected cancer cells (Figure 8A). Interestingly, results showed that EA treatment inhibited the growth of cancer cells in a dose-dependent manner, with IC_50_ values of 29.12 ± 1.15 μM and 20.51 ± 1.22 μM, respectively (Figure 8A). Cell viability assay indicated that EA significantly decreases the viability or proliferation of studied human breast cancer cell lines.

### 2.8. CDK6 Expression Studies

Enzyme inhibition and binding studies of EA with CDK6 suggested a high binding affinity and inhibition in the kinase activity. EA decreases the cell viability of breast cancer cells, so to confirm whether EA decreases the cellular expression of CDK6 or not, we have performed an expression study of CDK6 in EA-treated/control cells. Interestingly, we found that EA decreases the expression of CDK6 in MCF-7 and MDA-MB-231 cancer cells (Figure 8B). Figure S4 depicts uncropped images of membrane probed with CDK6 and actin antibodies after EA treatments. These results suggest that EA downregulates the expression of CDK6 at the translational level also and thus supported the results of enzyme inhibition and binding studies.

### 2.9. Colony Formation and Apoptosis Studies

CDK6 supported the colonization and apoptotic evasion of cancer cells [34]. Thus, CDK6 inhibition is an innovative strategy to decrease the colonization and apoptotic induction in cancer cells. We further analyzed the effect of EA on colony formation and apoptosis of MCF-7 and MDA-MB-231 cells. Results of colony formation studies showed that EA treatment decreases the number of colonies significantly, as compared to vehicle control (Figure 8C,D). Subsequently, we evaluated the apoptotic potential of EA in MCF-7 and MDA-MB-231 cells. The cells were treated with the respective IC_50_ concentration of EA for 48 h and processed for annexin-V staining and found that EA induces apoptosis in 23.3% and 27.9% of MCF-7 and MDA-MB-231 cells, respectively (Figure 8E). These observations of colony formation and apoptosis studies suggested that EA has a great potential to reduce the colonization of selected breast cancer cells and induces apoptosis.

## 3. Discussion

Most of the human cancers involve a deviation from normal signaling cascades [35,36]. Protein kinases govern the important steps of these signaling pathways and any unusual activation of the signaling cascades can be controlled by targeting these kinases [15,37]. Thus, at present, this domain has attracted the interest of researchers across the globe and anticancer therapeutics is largely devoted to the identification of molecules targeting these kinases [38,39,40]. In search of the development of therapeutic molecules against these kinases, natural products are at the heart of this due to their wide therapeutic potential along with minimum side effects. Natural products demonstrate the wealthy origin of novel molecular scaffolds for synthetic chemists who deploy these for the development of drugs and drug leads [41]. Thus, owing to all these properties, these compounds have been used as leads for drug discovery. Since ancient times, many phytochemicals or plant-based products have been effective to treat a wide variety of human disorders. This is attributed to the fact that these natural compounds possess broad-spectrum properties namely antioxidant [42], anti-inflammatory [43], anti-cancer activities, and many more. Thus, considering all these important properties of plant-based products, this study investigates the potential inhibitors of CDK6.

This study screens a series of plant-derived natural compounds against CDK6 to find a potent inhibitor of CDK6 that can be used as a drug lead in CDK6 directed cancers. Initially, CDK6 was cloned, expressed, and purified. After screening through molecular docking and fluorescence-based binding study, EA was selected for detailed analysis. EA is a dietary polyphenol widely found in fruits and vegetables and has well-established anti-cancerous properties [44]. EA is a pharmacologically significant polyphenol that is a promising compound in anticancer therapy [45]. Molecular docking of EA with CDK6 provided an insight into the binding pattern highlighting that EA binds to the ATP binding pocket of CDK6-forming covalent interactions with functionally important residues of CDK6. Additionally, molecular dynamics simulation of 200 ns was performed to have an atomistic detail of binding of EA to CDK6. Binding of EA to CDK6 induces a closed conformation of CDK6, which is implicated from the computed distance of 0.15–0.3 nm coupled with prevailing hydrogen bonds between the CDK6 and EA during MD simulations. Further, the stability of the system was also implicated from calculated *R*_g_ and RMSD values, which suggested the achievement of equilibration after 120 ns. The variation in *R*_g_ and RMSD values suggested that EA-bound CDK6 is slightly less stable as compared to free CDK6. Besides, the flexibility of the conformational states of free CDK6 and EA-bound CDK6 was analyzed using PCA which also showed that EA-bound CDK6 occupies a larger conformational space as compared to free CDK6. Further, free energy landscapes of both the conformations also implied that binding of EA to CDK6 perturbs its folding pattern, thereby inhibiting the functionality of the protein.

Fluorescence quenching studies suggested that EA binds to CDK6 with excellent affinity. Moreover, fluorescence spectroscopy carried out as a function of temperature provided an insight into the operative quenching mode of CDK6-EA interaction. The variation in binding parameters with temperature suggested that CDK6-EA quenching is guided by a mixture of static and dynamic modes of quenching. Thermodynamic analysis of CDK6-EA interaction showed Δ*H*° and Δ*S*° values in the positive range, thereby suggesting that prevalent forces driving this reaction were hydrophobic interactions. ITC further validated strong binding between CDK6 and EA with obtained binding parameters of significant values. ATPase activity of EA suggested it to be a potent inhibitor of CDK6 with an admirable IC_50_ value of 3.053 µM.

The success of therapeutic molecules in anticancer therapy also ascertains from their capability to selectively kill cancer cells without showing any cytotoxicity. Thus, a cell viability assay was carried out to see the effect of EA on breast cancer cells. Cell viability assay suggested that EA meaningfully decreases the growth of human breast cancer cells in the submicromolar range. These studies are also in close agreement with previous reports, which suggested that EA treatment decreases the growth of different human cancer cells [29,44]. Further, expression study of CDK6 in EA-treated/control cells indicated that EA decreases the expression of CDK6 in MCF-7 and MDA-MB-231 cells implying that along with the activity inhibition, EA also decreases the expression of CDK6 at the translational level. These observations validate our enzyme assay results and binding study observations. Further, colony formation and apoptosis suggested that EA is a potent compound that can reduce the colonization of selected breast cancer cells and induces apoptosis. Apoptosis studies are also in close agreement with some previous studies, which suggested that EA induces apoptosis in A549 cells via phosphoinositide 3-kinase/protein kinase B pathway. In addition to these reports, our observations suggested that EA could also induce apoptosis in a CDK6-dependent manner. Overall, the present study provides a different and novel insight into the possible targets of EA, particularly in cancer cells where the expression of CDK6 is high. The outcomes of this study also warrant a further detailed therapeutic evaluation of EA concerning the CDK6 targetted therapies.

## 4. Materials and Methods

### 4.1. Chemicals and Reagents

The gene for *CDK6* was obtained from Harvard Medical School and subsequently sub-cloned in the prokaryotic expression vector; pET-28a^+^ plasmid (Novagen, WI, USA). For cloning purposes, the DH5α strain of *E. coli* was used while BL21 (codon^+^) strain was used as the expression of recombinant CDK6 protein. Using the previous protocol, plasmid isolation, restriction enzyme digestion, ligation, and competent cell preparation was performed [18]. DifcoTM LB broth Miller (Fisher Scientific, Lenexa, KS, USA) for bacterial culture was from Becton, Dickinson, and Company, MD, USA. Annexin-V/PI staining kit was procured from BD-Biosciences (San Jose, CA, USA). Reagents for cell proliferation study were procured from Thermo Fisher Scientific (Waltham, MA USA). Breast cancer lines, MCF-7, and MDA-MB-231 were borrowed from National Centre for Cell Sciences (Pune, India).

### 4.2. Cloning, Expression, and Purification of CDK6

The 981-bp coding region (326 amino acids) was amplified by PCR from plasmid pcDNA containing the *CDK6* gene including the restriction sites *Nco*I and *Xho*I on each end. The *CDK6* gene was ligated into pET28a^+^ vector digested with a similar restriction enzyme to generate complementary sticky ends. The ligated product was verified using restriction digestion method. Finally, the orientation and gene sequence ligated into the expression vector was verified using gene sequencing.

Using a standard protocol, the constructed expression vector (pET28a+) comprising the coding region of the CDK6 was transformed into *E. coli BL21* (codon^+^) and selected using kanamycin. The overnight bacterial culture of the expression cells was transformed and CDK6 expression was induced by the IPTG. Once culture was full-grown, subjected to centrifugation, lysis, and obtained supernatant and pellet were checked on sodium dodecyl sulfate-polyacrylamide gel electrophoresis (SDS-PAGE) to confirm the presence of protein in the soluble or insoluble fraction.

The major portion of CDK6 was found in the inclusion bodies (IBs). To purify CDK6 from IBs we followed standard procedure and solubilized IBs were centrifuged and the supernatant was loaded on preequilibrated Ni-NTA Column (50 mM Tris buffer pH 8.0, 200 mM NaCl and 0.3% N-lauroylsarcosine and 20 mM imidazole). After the binding step, the column was washed, and then bound CDK6 was eluted with the help of elution buffer (50 mM Tris buffer pH 8.0, 200 mM NaCl, and 0.1% N-lauroylsarcosine, 300 mM imidazole). The fractions of CDK6 were collected and checked for purity by SDS-PAGE. The purified CDK6 was refolded by dialysis and refolded protein was used for all biochemical studies after checking its kinase activity. Western blot was done to confirm the CDK6 after transferring bands of protein from SDS-PAGE to the PVDF membrane. Blot was developed using diaminobenzidine or luminol method [46].

### 4.3. Molecular Docking

The atomics coordinates of the CDK6 structure were downloaded from the Protein Data Bank (PDB ID - 3NUP) and subsequently optimized for modeling the gap in the structures using the “PRIME” module of Schrödinger [47,48]. The structure of EA was constructed using drawing utilities present in MAESTRO (Maestro, Schrödinger, LLC, New York, NY, 2018) and the geometries of the resultant ligand structure were optimized by JAGUAR [49].

The Autodock 4 was used to perform the docking between the CDK6 and EA [50], generated output in the form of inhibition constant along with the free energy of binding. The Autodock performs rigid docking, which involves the usage of free energy factors for the classification of bound conformation. The energy factors are derived from the combination of the available empirical force field as well as the Lamarckian Genetic Algorithm [50]. In the primary step, the grid dimensions were set to 44 × 54 × 50 Å along with the XYZ directions using the AutoGrid with a spacing of 0.375 Å. The maximum efficiency values were set for the Lamarckian genetic algorithm, with the population control was set to 250 as well as the “longer” intervals, which were used for the energy evaluations. The docking was performed on the cluster of Center for High-Performance Computing (CHPC), South Africa, and 100 bound conformations which were grouped based on 2.0 Å RMSD tolerance were generated for CDK6 and EA system. The DrugScoreX was used for performing the re-scoring of the generated docked conformations [51]. The best scoring docked complex was subjected to the MD simulations.

### 4.4. MD Simulations

MD simulations on EA-bound and apo form of CDK6 were performed using GROMACS version 2018-2 [52]. Primarily, the GROMOS96 53a6 force-field was used for the generation of topologies of protein structure in the docking-based generated complexes. Moreover, the topologies of the studied ligand compound were generated using the PRODRG server [53]. But the PRODRG server does not contain the functionality of generating the partial charges of the EA, therefore, the CHELPG program and B3LYP 6-31G (d,p) basis set present in the DFT method of GAUSSIAN was used for the correction of the partial charges [54]. In the subsequent steps, the solvation of the docked complexes was performed using the SPC/E water model [55] and neutralization was performed by including the counter number of NA and CL ions. Furthermore, the steepest descent algorithm present in the GROMACS was used for minimizing the neutralized solvated system with a convergence criterion of 0.005 kcal/mol. To keep the ligand molecules in the solvated box, the position restraints were applied.

The equilibration step includes a separate NVT (constant volume) stage followed by NPT (constant pressure) ensemble, each at a 100 ps time scale. Using the Berendsen weak coupling method and Parrinello-Rahman barostat, the temperature and the pressure of the simulating system were maintained at 300 K and 1 bar, respectively. The LINCS algorithm was used for the generation of the final conformational production stage for a 200 ns timescale, and trajectories were generated, which were analyzed to understand the behavior of each complex in the explicit water environment. The changes in the protein-ligand distance, H-bonds, RMSD, *R*_g_, RMSF, PCA, and free energy landscapes of the complex system were analyzed. Furthermore, the molecular mechanics Poisson-Boltzmann surface area (MM-PBSA) protocols implemented in the g_mmpbsa package [56] were used for the calculation of free energy of binding protein and the ligand molecules.

### 4.5. Enzyme Inhibition Assay

ATPase assay was performed to see the CDK6 inhibitory activity of EA [57]. For a typical kinase reaction, freshly prepared ATP (50 µM) was incubated with CDK6 (1 µM) in a reaction volume of 100 μL and incubated at 25 °C for 1 h. Similar reactions were set up with the increasing concentration of EA. Malachite green (200 μL) was further added to the reaction mixture to stop the reaction followed by incubation of samples at room temperature for 20–25 min for the development of color. The absorbance of the final reaction product was measured spectrophotometrically at 620 nm.

### 4.6. Fluorescence Measurements

To study the binding affinity of EA with the recombinant CDK6, a fluorescence binding study was performed. CDK6 was titrated with the EA and corresponding emission spectra were recorded in the range of 300–400 nm after carrying out excitation at 280 nm. The inner filter effect [58] was corrected for the obtained intensities. Fluorescence emission spectra were analyzed by the Stern-Volmer equation to find Stern-Volmer constant (*K_sv_*) and modified Stern-Volmer equation to estimate the binding constant (*K*) and the number of binding sites (*n*). Other binding features were estimated by our previously described protocol [40,59].

### 4.7. Thermodynamics of the Complex

With each reaction, changes in enthalpy and entropy are associated, which are evidence of the type of reaction taking place. Equation (1) (*“van ’t Hoff equation”*) [23] was employed to calculate the thermodynamic parameters associated with this binding,

(1) ΔG°=−RTLnK=ΔH°−TΔS°

“*K* is the obtained binding constant.

“Δ*H*° is the associated enthalpy change while ∆*G*° is the associated free Gibbs energy change”.

*Δ*S*° denotes the associated entropy change with reaction and R is “universal gas constant” (1.987 cal mol^−1^·K^−1^).

### 4.8. Isothermal Titration Calorimetry

ITC measurements were carried out at 25 °C using a VP-ITC microcalorimeter (MicroCal, Inc, GE, MicroCal, USA). The sample cell was filled with 15–20 µM CDK6 and 500 µM EA solution was filled in the syringe. An automated titration was carried out with (including a first false injection of 2 μL) a successive injection of 10 μL EA solution into a CDK6 sample cell at a 260 s interval with 320 rpm stirring speed. For analysis, the heat of dilution of EA in the sample buffer was subtracted from the titration data. MicroCal Origin 8.0 was used to analyze the stoichiometry of binding (*n*), enthalpy change (Δ*H*), an association constant (*K*_a_).

### 4.9. Cell Viability Assay

Cell viabilities of cancer cells with or without EA were evaluated using MTT assay [28,60]. Briefly, the selected cells (MCF-7 and MDA-MB-231) were seeded in a 96-well (5000–6000 cells/well) culture plate. Overnight grown cells were incubated with EA (0–250 μM) for 72 h in a CO_2_ incubator, at 37 °C. After a stipulated time of treatment, 20 µL of MTT (from 5 mg/mL stock solution in PBS, pH 7.4) was added to each treatment well of 96-well plate, and plates with MTT solution were incubated additionally for 4–5 h, in the CO_2_ incubator. Lastly, the formazan were solubilized by adding 100 µL of DMSO to each well. The absorbance (A) of the dissolved purple reaction solution was measured at 570 nm. Absorption readings were converted into percent cell viability and used to estimate 50% inhibitory concentration (IC_50_) of EA for selected cancer cells.

### 4.10. Total Protein Isolation and Immunoblotting

The cell lysates of EA-treated/untreated MCF-7 and MDA-MB-231 cell lines were prepared using RIPA cell lysis buffer (Thermo Fisher Scientific (USA)) and total protein was isolated. The amount of protein was quantified by a BCA-protein estimation kit. Nearly, 50–60 μg of total protein was subjected for SDS-PAGE, and obtained bands were blotted to polyvinylidene fluoride membrane. The protein of interest was identified using respective primary antibodies and horseradish peroxidase (HRP) conjugated secondary IgG using the luminol method [46].

### 4.11. Colony Formation Assay

To perceive the effect of EA treatment on the colonogenic potential of selected cell lines colony formation studies were performed as described previously [28]. Briefly, ~1500–2000 (MCF-7/MDA-MB-231 cells) cells/well were seeded (in triplicates) in each well of a 6-well cell culture plate. The cells were grown in complete cell growth medium for 48 h and incubated with respective IC_50_ concentrations of EA for 10–12 days, (at 37 °C, in a 5% CO_2_ incubator). On the other hand, the control cells were incubated with vehicle control (DMSO) under similar experimental conditions. After 10–12 days, the colonies of cells were fixed with 100% methanol and subsequently stained using a 0.4% crystal violet solution (prepared in 25% methanol. Finally, the colonies obtained after staining were photographed, quantified, plotted, and analyzed by comparing with vehicle control.

### 4.12. Apoptosis Assay

EA was evaluated for its apoptosis-inducing potential on selected cancer cell lines using Annexin-V staining [61]. In brief, cells were plated in a six-well culture plate, and once they reached ~70%–80% confluency, incubated with IC_50_ concentration of EA or vehicle control for 24 h at 37 °C. Following the incubation with EA (24 h), cells were collected, washed two times with PBS, and incubated with Annexin-V/PI stain and analyzed by Flow Cytometry as per manufacturer’s protocol (Annexin-V kit, BD-Biosciences, San Jose, USA).

### 4.13. Statistical Analysis

All the experiments were performed at least three times and their average was taken and expressed in mean ± standard error of the mean (SEM).

## 5. Conclusions

The aberrant activation of signaling cascade is a frequent event in various types of human cancers. CDK6 is directly associated with the progression of different types of cancer because the overexpression of CDK6 resulted in an uncontrolled cell cycle progression and growth. For the identification of therapeutic molecules, natural compounds serve as the source of enormous structural and chemical diversity along with diverse biological activities with fewer side effects. This study identifies EA as a potent inhibitor of CDK6. However, the anticancer properties of EA were reported earlier, but the present study establishes CDK6 as a novel target. Thus, targeting CDK6 by EA can be a smart therapeutic approach to manage CDK6 directed cancers.

## Figures and Tables

**Table 1:** Thermodynamic parameters of CDK6-EA obtained through a Stern-Volmer plot.

Temperature (K)	*K_sv_* (10^4^ M^−1^)	*K_q_* (10^12^ M^−1^s^−1^)	R^2^
298	5.8	5.8	0.97
301	14.2	14.2	0.99
303	19.8	19.8	0.98

**Table 2:** Thermodynamic parameters of CDK6-EA interaction.

Temperature (K)	*K* (10^7^ M^−1^)	*n*	Δ*G*° (kcal mol^−1^)	Δ*S* (cal.mol^−1^K^−1^)	Δ*H*° (kcal mol^−1^)	TΔS° (kcal mol^−1^)
298	0.29	1.32	−8.73	283.9087	75.8657	84.60
301	0.65	1.16	−9.59			85.45
303	2.6	1.41	−10.16			86.02

## Supplementary Materials

Supplementary materials can be found at https://www.mdpi.com/1422-0067/21/10/3526/s1.
