# Supplementary material for: Ellagic Acid Controls Cell Proliferation and Induces Apoptosis in Breast Cancer Cells via Inhibition of Cyclin-Dependent Kinase 6
Source: Int J Mol Sci. 2020 May 15;21(10):3526. doi: 10.3390/ijms21103526 (PMC7278979; doi:10.3390/ijms21103526)
Supplement: Supplementary file 1 [file ijms-21-03526-s001.pdf]

Supplementary Materials

Ellagic Acid Controls Cell Proliferation and Induces Apoptosis in Breast Cancer Cells via inhibition of Cyclin-Dependent Kinase 6

Mohd Yousuf[1], Anas Shamsi[2], Parvez Khan[2], Mohd Shahbaaz[3,4], Mohamed F. AlAjmi[5], Afzal Hussain[5], Gulam Mustafa Hassan[6], Asimul Islam[2], Qazi Mohd Rizwanul Haque[1] and Md. Imtaiyaz Hassan[2,*]

[1]Microbiology Research Laboratory Department of Biosciences, Jamia Millia Islamia, Jamia Nagar, New Delhi 110025, India
[2]Centre for Interdisciplinary Research in Basic Sciences, Jamia Millia Islamia, Jamia Nagar, New Delhi 110025, INDIA.
[3]South African Medical Research Council Bioinformatics Unit, South African National Bioinformatics Institute, University of the Western Cape, Private Bag X17, Bellville, Cape Town 7535, South Africa
[4]Laboratory of Computational Modeling of Drugs, South Ural State University, 76 Lenin Prospekt, Chelyabinsk, Russia, 454080.
[5] Department of Pharmacognosy College of Pharmacy, King Saud University, Riyadh 11451 KSA.
[6]Department of Biochemistry, College of Medicine, Prince Sattam Bin Abdulaziz University, P.O. Box 173, Al-Kharj – 11942, Kingdom of Saudi Arabia.

To whom all correspondence should be addressed,

Md. Imtaiyaz Hassan, Ph.D., FRSB, FRSC.
Centre for Interdisciplinary Research in Basic Sciences
Jamia Millia Islamia, Jamia Nagar
New Delhi 110025, INDIA
Cell: +91-9312812007
E-mail: mihassan@jmi.ac.in

Table S1. Binding parameters of all the screened natural compounds with CDK6 obtained from molecular docking and fluorescence binding studies.

S. No.	Compounds	Structure	ΔG # (kcal/mol)	Binding constant* (K) M^{-1}
1.	Caffeic Acid	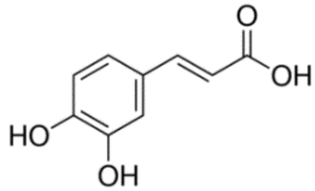	–6.8	1.1 X 10^4
2.	Ferulic Acid	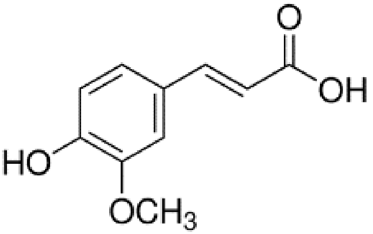	–6.8	0.76 X 10^3
3.	Urosolic acid	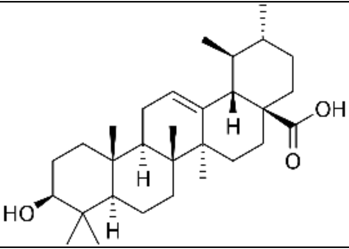	–5.4	0.64 X 10^1
4.	**Ellagic Acid**	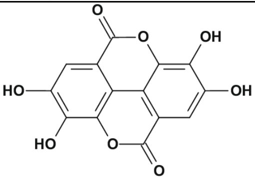	**–7.9**	**2.6 X 10^7**
5.	Rosmarinic acid	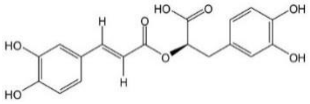	–7.7	1.19 X 10^4
6.	Capsaicin	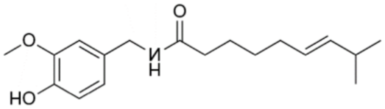	–6.7	NA
7.	Tocopherol	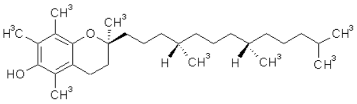	–7.6	NA
8.	Limonene	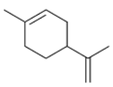	–6.3	NA

#Binding affinity of the selected compounds with CDK6 predicted through Molecular docking. *Binding constant calculated from fluorescence studies. Binding constant values could not be predicted in some cases and mentioned as not applicable (NA).

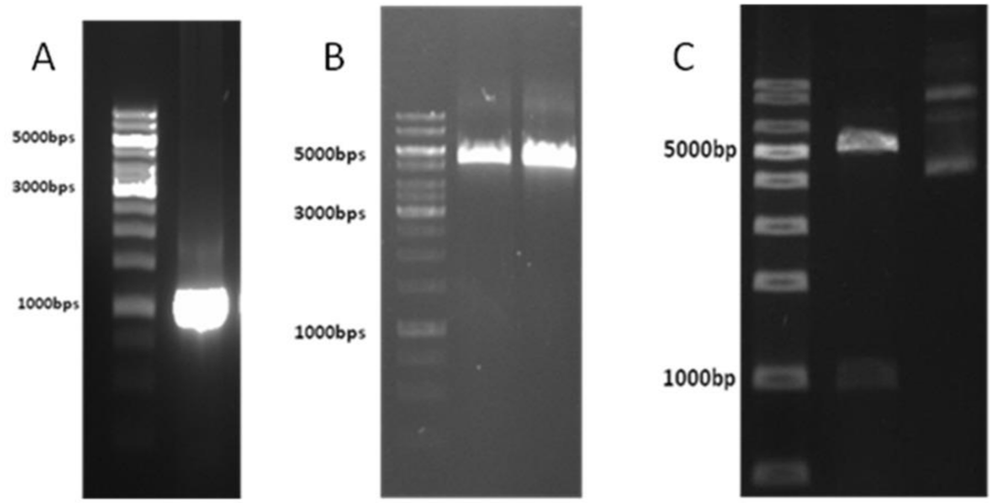

Figure S1. Cloning of CDK6 gene: (A) Amplified CDK6 gene. Lane 1: Marker and Lane 2: amplified product of CDK6 gene, **(B)** Digested pET28a plasmid. **(C)** Confirmed constructed plasmid by colony PCR. Lane 1: marker, Lane 2: digested CDK6 with pET28a, Lane3: pET28a plasmid with CDK6.

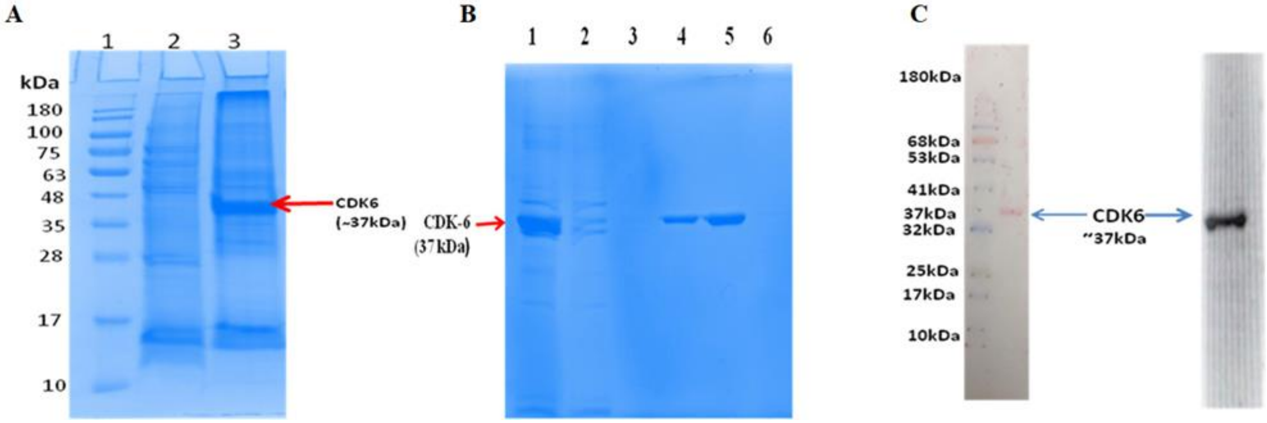

Figure S2. Expression and purification of recombinant CDK6: (A) Expression of CDK6 protein. Lane 1: Marker, Lane 2: Uninduced CDK6 sample, Lane 3: Induced CDK6 sample; **(B)** Purification profile of CDK6. Lane 1: Before binding, Lane 2: After binding, Lane 3: 20 mM Imidazole, Lane 4: 100 Mm Imidazole, Lane 5: 250 mM Imidazole, Lane 6: 500 mM Imidazole; **(C)** Western blot of purified His-tag CDK6 protein.

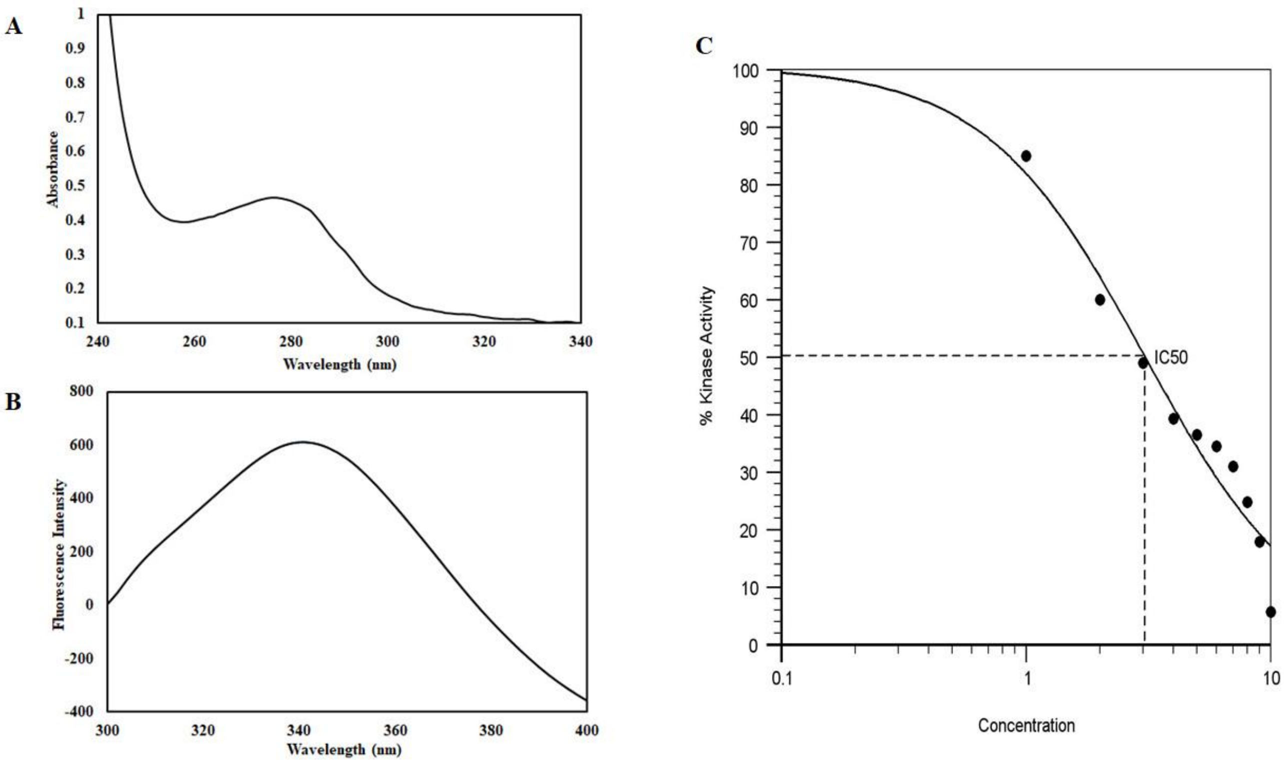

Figure S3. (A) UV Absorption spectra of purified CDK6 in the range of 240-340 nm. **(B)** Fluorescence spectra of purified CDK6. **(C)** IC_{50} plot obtained through the AAT Bioquest calculator [67].

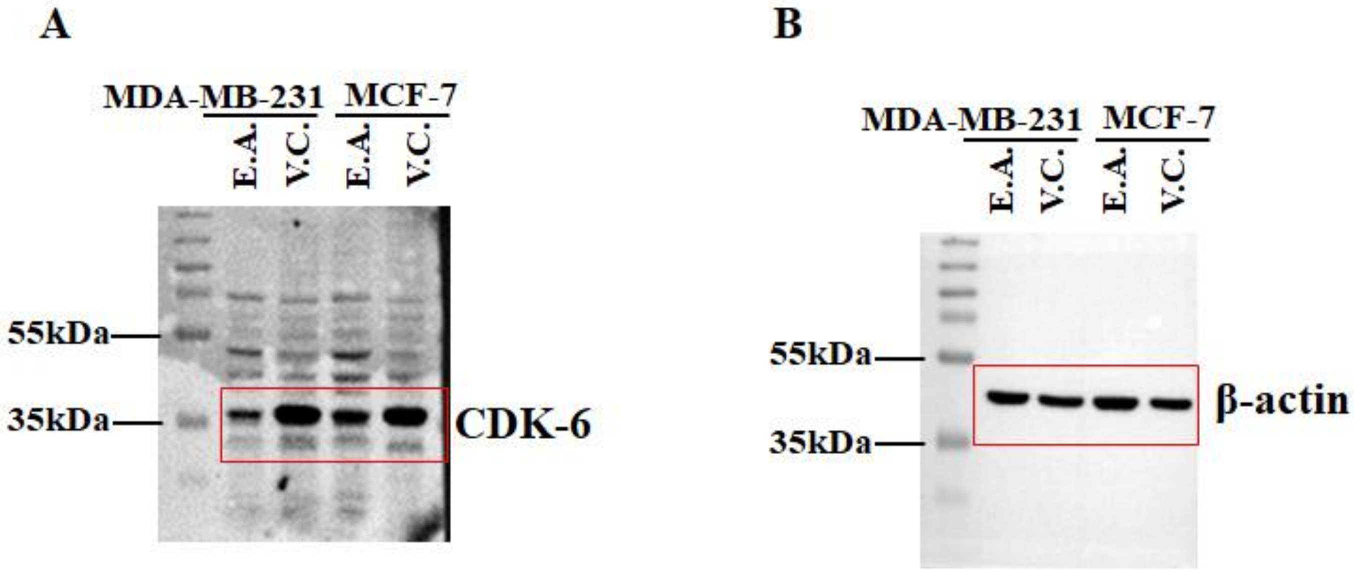

Figure S4. Uncropped images of membrane probed with CDK6 and actin antibodies after EA treatments.
